# A large-scale perspective for managing prairie avifauna assemblages across the western US: influences of habitat, land ownership and latitude

**DOI:** 10.7717/peerj.2879

**Published:** 2017-01-18

**Authors:** Victoria J. Dreitz, Lani T. Stinson, Beth A. Hahn, Jason D. Tack, Paul M. Lukacs

**Affiliations:** 1 Department of Ecosystem and Conservation Sciences, Wildlife Biology Program and Avian Science Center, College of Forestry and Conservation, University of Montana, Missoula, MT, United States; 2 Department of Ecosystem and Conservation Sciences, Wildlife Biology Program, College of Forestry and Conservation, University of Montana, Missoula, MT, United States; 3 Department of Fish, Wildlife and Conservation Biology, Colorado State University, Fort Collins, CO, United States; 4 US Department of Agriculture—Forest Service, Northern Region, Missoula, MT, United States; 5 Aldo Leopold Wilderness Research Institute, Missoula, MT, United States; 6 US Department of Agriculture, Sage Grouse Initiative, Natural Resources Conservation Service, Missoula, MT, United States

**Keywords:** Community structure, Grassland obligates, Land ownership, Large-scale, Prairie avifauna, Sagebrush obligates, Species richness

## Abstract

Future demands for increased food production are expected to have severe impacts on prairie biodiversity and ecosystem integrity. Prairie avifauna of North America have experienced drastic population declines, prompting numerous conservation efforts, which have been informed primarily by small-scale studies. We applied a large-scale perspective that integrates scale dependency in avian responses by analyzing observations of 20 prairie bird species (17 grassland obligates and three sagebrush obligate species) from 2009–2012 in the western prairie region of the United States. We employed a multi-species model approach to examine the relationship of land ownership, habitat, and latitude to landscape-scale species richness. Our findings suggest that patterns and processes influencing avian assemblages at the focal-scale (e.g., inference at the sampling unit) may not function at the landscape-scale (e.g., inference amongst sampling units). Individual species responses to land ownership, habitat and latitude were highly variable. The broad spatial extent of our study demonstrates the need to include lands in private ownership to assess biodiversity and the importance of maintaining habitat diversity to support avian assemblages. Lastly, focal-scale information can document species presence within a study area, but landscape-scale information provides an essential complement to inform conservation actions and policies by placing local biodiversity in the context of an entire region, landscape or ecosystem.

## Introduction

Global loss of biological diversity is occurring at unprecedented rates across numerous taxa and habitats with serious repercussions for ecosystem health and human well-being. Biodiversity loss can alter key ecological processes, affect ecosystem productivity and stability, and impact societally-valuable ecosystem services (Hooper et al., 2005; Tilman, Reich & Knops, 2006; Cardinale et al., 2012). While the specific factors leading to species extinctions are numerous and complex, the primary driver over the last century is human-caused land use change (Vitousek et al., 1997). Finding solutions to conserve declining populations is one of the most important tasks currently facing ecologists, land managers, and policymakers.

Temperate grasslands and shrublands (hereafter prairies) have undergone extensive conversion because of their global value for human food production. Prairies remain some of the world’s least protected habitats (Hoekstra et al., 2005) and within the western region of North America are among the most imperiled ecosystems (Samson & Knopf, 1994; Knick et al., 2003). Over the last two centuries, replacement of native grazers with domestic livestock, fire suppression, agricultural expansion, and infrastructure development have profoundly impacted North American prairies. These impacts influence ecological processes and functions important to wildlife, such as resource selection, community interactions and reproductive performance (Samson & Knopf, 1994), resulting in declines in species diversity and ecosystem health. Land alteration for agricultural, residential, and industrial needs is linked to land ownership patterns (Lovett-Doust et al., 2003) but only a few studies have considered the association of land ownership to differences in biodiversity (e.g., Lovett-Doust & Kuntz, 2001; Lovett-Doust et al., 2003; Ortega-Huerta & Kral, 2007). Patterns of biodiversity on private prairie lands are particularly relevant to conservation planning and policy-making decisions, as a growing global demand for food production will disproportionately be met in privately-owned prairie landscapes.

Some of the most dramatic and consistent declines in prairie wildlife have been documented in the avifauna. In North America, prairie birds have undergone greater declines than any other avian assemblage (Knopf, 1994; Knick et al., 2003; Askins et al., 2007). Numerous studies on prairie avifauna habitat associations have been undertaken at local sites (Knick et al., 2003; Askins et al., 2007). These analyses identify factors influencing populations locally, and these findings are incorporated into larger-scale conservation and management plans. The consequences of focal-scale conservation efforts are largely unknown but may include unmet conservation goals resulting from failed integration of ecosystem patterns and processes, especially in fragmented landscapes (Hobbs & Norton, 1996) such as prairies (Baudron & Giller, 2014). The importance of scale in the resolution of geographic patterns observed by species has long been recognized (Johnson, 1980). We refer to scale as the geographic space where focal-scale is the area represented by each data point and landscape-scale is inference to which the entire set of sampling units applies (Rahbek, 2005). A large-scale perspective is essential to identify avian habitat use patterns to best inform conservation efforts.

We seek to examine the hypothesis that landscape-scale characteristics of vegetation, land tenure, and latitude drive the richness of prairie avifauna occurrence. We predict that areas containing high percentages of grassland habitats will have higher diversity of grassland species and areas with high percentages of sagebrush habitats will have higher diversity of sagebrush species. Privately-owned lands are under greater economic pressure than public lands in satisfying human needs for goods and services (Daily, 1997; Vitousek et al., 1997). Thus, we predict that lands in public ownership will support higher diversity than lands in private ownership (e.g., agriculture and private lands). Lastly, studies on latitudinal gradients in species richness supports a near-universal pattern of an increasing richness from polar to equatorial regions (MacArthur & Wilson, 1963; Hawkins & Porter, 2001; Hillebrand, 2004). We predict a similar relationship between latitude and prairie avifauna species richness.

Here, we assess how land ownership, habitat, and latitude influence prairie bird distributions across the western prairie region of the US. Our study differs from previous work by deriving estimates of species richness while accounting for imperfect detection. Other studies note the difficulties in comparing richness metrics spatially (Hurlbert & Haskell, 2003) and temporally (White & Hurlbert, 2010) because of variation in survey effort (one of many factors influencing detection probability). We use methods that explicitly partition heterogeneity from detection such that ecological inference is unbiased. This model-based approach estimates the occurrence probability of all species in a community by linking multiple single-species occurrence models into a single model, thus improving inferences on all species, especially those that are rare or observed infrequently (Zipkin et al., 2010). Our multi-species model improves precision of estimates for individual species while simultaneously examining associations of entire community assemblages (Royle & Dorazio, 2008; Dewan & Zipkin, 2010; Kéry, 2010; Zipkin, Dewan & Royle, 2009; Zipkin et al., 2010; Karanth et al., 2014). This robust characterization of the patterns and associations of prairie bird communities across a large geographic region provides powerful inference to inform local and regional conservation and management policies and initiatives.

## Materials & Methods

### Study area

**Figure 1:**
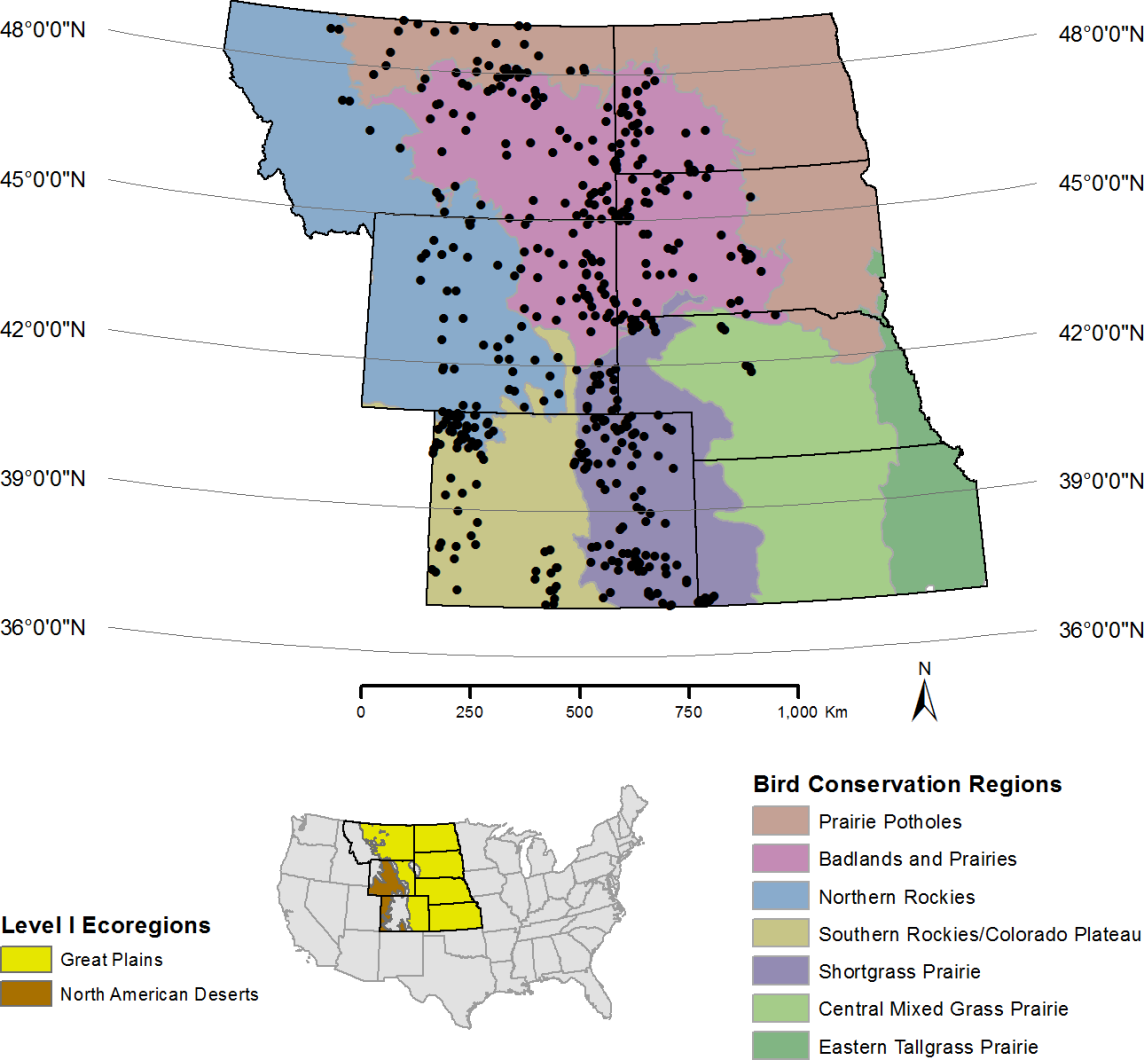
Map of the region in the western United States surveyed for prairie avifauna from 2009–2012 in relation to the Great Plains and North American Deserts ecoregions (lower left) and Bird Conservation Regions (in color). Circles represent the centroid of 1 km^2^ sampling plots (*n* = 413) that contained 16 point count stations evenly-spaced at 250 m.

The study area is approximately 140,000,000 ha within the prairie landscape of the western United States defined by the Great Plains and North American Deserts ecoregions (Commission for Environmental Cooperation (CEC), 1997; Fig. 1). This landscape is comprised of seven western US states and six Bird Conservation Regions (BCRs)—distinct ecological regions with similar biotic and abiotic characteristics and natural resource management issues (US North American Bird Conservation Initiative (US NACBI), 2000). Management of the lands within the study area is by private landowners (51.6%), US Department of Interior—Bureau of Land Management (BLM; 22.2%) and other federal agencies (e.g., US Department of Agriculture—Forest Service (USFS; 16.5%)), Native American tribes (2.8%), and US states’ management agencies (3.1%; Table 1).

**Table 1:** Environmental covariates used to distinguish avian responses at the focal-scale from landscape-scale population responses of grassland and sagebrush obligate avian species. Covariates were calculated at each sampling plot, 1 km^2^ square containing 16 evenly-spaced point count stations, and summed across a total of 413 sampling plots surveyed for avian species from 2009–2012.

Measures	Description	Values	
Land ownership		%	Hectares
	Public		
	Bureau of Land Management	22.2	9212.22
	US Forest Service	16.5	6824.82
	Bureau of Indian Affairs	2.8	1154.18
	State Managed Land	3.1	1277.89
	Other Federal (e.g., Dept. of Defense)	2.3	939.85
	National Park Service	1.0	396.28
	US Fish and Wildlife Service	0.6	263.91
	*Total Public*	*48.4*	*20069.16*
	Private	51.6	21403.72
Habitat		%	Hectares
	Grassland		
	Mixed Grass Prairie	27.1	11680.41
	Shortgrass Prairie	12.2	5154.50
	Sand Prairie/Sandhill Steppe	6.2	2690.87
	Montane and Foothill Shrubland and Grassland	1.1	654.96
	*Total Grassland*	*46.6*	*20180.74*
	Sagebrush		
	Sagebrush Shrubland	21.2	10042.34
	Steppe and Semi-desert Scrub Shrub	3.7	2277.88
	*Total Sagebrush*	*25.9*	*12320.22*
	Other (e.g., Wetlands and Open Water)	28.5	13776.35
Latitude (NAD83)		Degree Decimal	
	Average	42.69	
	Minimum	36.93	
	Maximum	48.95	

### Sampling: design-based

The bird data consisted of point count surveys collected from 2009–2012 as part of a regional, multi-agency landbird monitoring collaboration, the Integrated Monitoring in Bird Conservation Regions (IMBCR) project. This design-based program covers all land area within the monitoring landscape, regardless of land ownership, habitat, or other factors (e.g., roads, terrain). Bird surveys were conducted across areas designated by fixed attributes such as ownership boundaries, state boundaries or BCR boundaries (White et al., 2012), hereafter referred to as strata. Within each stratum, grid-scale sampling plots were selected using a generalized random tessellation stratified sample, a spatially-balanced sampling algorithm (Stevens & Olsen, 2004; Theobald et al., 2007). Each sampling plot measured 1 km^2^ in area (Fig. 1) and contained 16 bird point count stations evenly spaced at 250 m to form a 4 × 4 grid of points. Each 1 km^2^ sampling plot represented the grid-scale, while a single point count station within a given plot represented the point-scale. Observers conducted a 6-min survey at each point count beginning 30 min prior to sunrise and concluding prior to 11 AM (local time) once per year. For every bird visually detected, the species, horizontal distance to the detected bird and minute interval were recorded; however, we truncated data into detection/non-detection data. We overlaid land cover data from the USGS National Gap Analysis Program (US Geological Survey, Gap Analysis Program (US GAP), 2011) in ArcGIS 10 to select sampling plots containing prairie habitats (<30% Forested & Woodland; *n* = 413 sampling plots) for use in the analysis.

### Guild delineation

Patterns of prairie bird occurrence and habitat associations can be examined at various levels, ranging from the individual species to the species assemblage. The guild concept is one way of classifying species assemblages; however, “guild” is a term that lacks consistent definition in the literature (Verner, 1984; Szaro, 1986; Simberloff & Dayan, 1991). We defined a guild specifically as either grassland (*n* = 17 species; Vickery et al., 1999) or sagebrush (*n* = 3 species; Paige & Ritter, 1999; Hanser & Knick, 2011) obligates (Table 2). We explicitly chose obligates to reflect their specific habitat preferences as opposed to generalists which reflect a different set of processes. The 20 species included in our analyses are designated as a conservation priority (e.g., species petitioned for US federal listing; US state-level species of concern; USFS Management Indicator Species). We limited our analyses to passerines and upland shorebirds (i.e., *Charadridae*) detected ⩾ 10 times in ⩾ 1 year.

**Table 2:** Coefficient estimates (with 95% credible intervals) for covariate influence on occupancy prairie birds from multi-species model. Co-variates summarize the proportion of public ownership (Public), grassland (Grass) or sagebrush cover type (Sage), and the latitude of 1 km^2^ sampling plots. Estimates reflect data with scaled predictors (*μ* = 0, *σ* = 1). Each species was identified as belonging to a grassland (G) or sagebrush (S) guild.

Species	Public	Grass	Sage	Latitude	Int
Baird’s Sparrow (G)	0.07 (−0.26, 0.4)	0.42 (0, 0.84)	−0.08 (−0.68, 0.49)	2.24 (1.68, 2.86)	−4.63 (−5.47, −3.88)
Bobolink (G)	−0.25 (−0.52, 0.01)	0.34 (0.04, 0.65)	−0.65 (−1.18, −0.17)	1.4 (1.07, 1.75)	−3.41 (−3.86, −2.99)
Cassin’s Sparrow (G)	−0.11 (−0.37, 0.14)	0.39 (0.15, 0.64)	−1.7 (−3.12, −0.72)	−2.51 (−3.02, −2.05)	−4.43 (−5.39, −3.68)
Chestnut-collared Longspur (G)	0.06 (−0.19, 0.31)	0.96 (0.61, 1.32)	−0.08 (−0.49, 0.32)	2.17 (1.76, 2.61)	−3.66 (−4.2, −3.16)
Dickcissel (G)	0.31 (−0.07, 0.7)	−0.45 (−0.92, 0)	−1.65 (−2.79, −0.75)	0.15 (−0.29, 0.57)	−4.56 (−5.34, −3.92)
Grasshopper Sparrow (G)	0.29 (0.14, 0.44)	0.7 (0.53, 0.87)	−0.6 (−0.81, −0.4)	0.74 (0.59, 0.9)	−0.35 (−0.5, −0.2)
Horned Lark (G)	−0.22 (−0.37, −0.08)	0.49 (0.33, 0.65)	0.19 (0.03, 0.34)	−0.05 (−0.19, 0.09)	0.84 (0.7, 0.98)
Lark Bunting (G)	−0.4 (−0.54, −0.26)	0.85 (0.69, 1.01)	0.32 (0.17, 0.48)	0.06 (−0.08, 0.2)	−0.31 (−0.44, −0.17)
Long-billed Curlew (G)	−0.19 (−0.47, 0.08)	−0.3 (−0.65, 0.03)	−0.12 (−0.48, 0.24)	1.34 (1.01, 1.7)	−3.07 (−3.52, −2.67)
Marbled Godwit (G)	−0.22 (−0.56, 0.11)	0.02 (−0.39, 0.44)	−0.54 (−1.08, −0.04)	2.79 (2.16, 3.52)	−5.02 (−6, −4.19)
McCown’s Longspur (G)	−0.19 (−0.44, 0.05)	0.38 (0.1, 0.67)	−0.46 (−0.92, −0.06)	0.37 (0.12, 0.63)	−2.89 (−3.22, −2.59)
Mountain Plover (G)	0.11 (−0.27, 0.51)	0.43 (−0.14, 1.04)	−0.63 (−1.57, 0.17)	−0.88 (−1.66, −0.27)	−3.59 (−4.31, −2.9)
Savannah Sparrow (G)	−0.36 (−0.6, −0.13)	−0.15 (−0.43, 0.11)	−0.51 (−0.85, −0.18)	1.4 (1.12, 1.69)	−2.81 (−3.15, −2.5)
Sprague’s Pipit (G)	−0.15 (−0.54, 0.24)	0.69 (0.2, 1.2)	0.13 (−0.42, 0.66)	3.63 (2.7, 4.72)	−6.81 (−8.49, −5.44)
Upland Sandpiper (G)	0.14 (−0.06, 0.35)	0.73 (0.47, 1)	−0.07 (−0.34, 0.19)	1.38 (1.09, 1.71)	−1.85 (−2.09, −1.62)
Vesper Sparrow (G)	−0.1 (−0.25, 0.06)	−0.02 (−0.19, 0.16)	0.96 (0.77, 1.16)	0.83 (0.66, 1)	−0.24 (−0.39, −0.08)
Western Meadowlark (G)	−0.26 (−0.5, −0.02)	0.98 (0.62, 1.38)	−0.09 (−0.32, 0.13)	0.44 (0.18, 0.72)	2.99 (2.66, 3.38)
Brewer’s Sparrow (S)	0.02 (−0.14, 0.17)	0.08 (−0.1, 0.27)	1.06 (0.88, 1.25)	0.05 (−0.11, 0.21)	−0.76 (−0.92, −0.61)
Sage Sparrow (S)	0.72 (0.41, 1.05)	−1.33 (−2.31, −0.46)	0.81 (0.57, 1.05)	−1.06 (−1.43, −0.69)	−4.1 (−5.08, −3.24)
Sage Thrasher (S)	0.81 (0.54, 1.1)	−1 (−1.46, −0.59)	0.67 (0.46, 0.89)	−0.68 (−0.98, −0.4)	−3.2 (−3.64, −2.79)

### Environmental covariates

We examined the relationship of land ownership, habitat and latitude to regional species richness of grassland and sagebrush obligate avifauna. For each of the 413 sampling plots, we calculated the percent area covered by each type of land ownership (private and public) and habitat (grassland, sagebrush, and other). Public ownership was defined as lands managed by US federal or state agencies. We also included lands managed by North American tribes in the public ownership category, given collective management strategies for many stakeholders. Private ownership included all lands not in public ownership. Habitat was assessed by calculating the percentage of grassland and sagebrush within each sampling plot was based on the National Vegetation Classification (NVC) (i.e., Class/Subclass, Form) and the Ecological Systems Classification (US Geological Survey, Gap Analysis Program (US GAP), 2011). Grassland was defined by four NVC Ecological Systems: (1) mixed grass prairie, (2) shortgrass prairie, (3) sand prairie/sandhill steppe and, (4) montane and foothill shrubland and grassland. Sagebrush was defined by two NVC Ecological Systems: (1) sagebrush shrubland and (2) steppe and semi-desert scrub shrub. Additional habitats in the sampling plot—cultivated cropland and pastures, wetland and open water, forest and woodlands, and developed—were grouped as ‘other’ habitat. Lastly, we used the centroid of each sampling plot to calculate latitude. The latitudinal gradient theory of poleward biodiversity decline is one of the longest recognized (MacArthur & Wilson, 1963), albeit not completely understood, patterns in ecology (Hillebrand, 2004; Mittelbach et al., 2007; Salisbury et al., 2012). Studies conducted at multiple scales, on many taxa and across different biogeographic regions have proposed numerous explanations for this gradient (Hillebrand, 2004; Mittelbach et al., 2007; Salisbury et al., 2012; Karanth et al., 2014). Latitude functions as a surrogate for a suite of environmental factors, thus, interlinking and confounding the distinct underlying hypotheses (Rahbek, 2005). Therefore, using latitude as a predictor allowed us to make inference on covariates of interest, while reducing the parameter space necessary to explain variation in avian occurrence.

### Analysis: model-based

We used a multi-species occupancy/richness hierarchical model as described by Dewan & Zipkin (2010), Zipkin, Dewan & Royle (2009) and Zipkin et al. (2010) to estimate the number of species within each guild present at each sampling plot. We combined individual species occurrence models into a single model by assuming that species covariate effects arise from a common distribution, allowing for more precise estimates of occupancy (Zipkin, Dewan & Royle, 2009; Kéry, 2010). In the context of estimating occupancy/richness, hierarchical models can distinguish absence from non-detection by incorporating models that specify presence versus absence as one process and detection versus non-detection as another process (Zipkin, Dewan & Royle, 2009).

For species-level models, we assumed that the the latent state of occurrence for a given species *z*_*i*_, is a Bernoulli process where the probability that species *i* is present at sampling plot *j*(*z*_*i*,*j*_ = 1) is *ψ*_*i*,*j*_ (MacKenzie & Kendall, 2002). We modeled species (*i*) and site (*j*) level heterogeneity in occurrence probabilities using a logit link function with relevant covariates such that:

\begin{eqnarray*}logit \left( {\psi }_{ij} \right) ={\alpha }_{0,i}+{\alpha }_{1i}{PUBLIC}_{j}+{\alpha }_{2i}{GRASS}_{j}+{\alpha }_{3i}{SAGE}_{j}+{\alpha }_{4i}{LAT}_{j}+{\tau }_{i}, \end{eqnarray*}

where *α*_0_ is the intercept and *α*_1_ − *α*_4_ are the effects of the environmental covariates on species *i* and *τ*_*i*_ is a random effect for species *i*. We used covariates on the percent of grassland (GRASS) and sagebrush (SAGE) habitat along with public land ownership (PUBLIC) and latitude (LAT).

Because species are imperfectly detected during sampling (MacKenzie & Kendall, 2002), we assumed that true occurrence, (z_*i*,*j*_ = 1), is a latent process that is only partially observable. If an observer detected species *i* at sampling plot *j* during point count *k*, denoted *x*_*i*,*j*,*k*_ = 1, then it can be determined that z_*i*,*j*_ = 1. However, if a species is not detected it could be that the species was either absent or undetected during sampling.

We assumed detection probability (*p*_*i*_) of species *i* was unlikely to vary by land ownership. However, landcover is likely to influence variation in species detectability through vegetative structure and available habitat, so we included covariates for grassland and sagebrush cover and included latitude to incorporate other unmeasured sources of variation in species specific models:

\begin{eqnarray*}logit \left( {p}_{i,j} \right) ={\beta }_{0,i}+{b}_{1,i}GRAS{S}_{j}+{b}_{2,i}SAG{E}_{j}+{b}_{3,i}LATITUD{E}_{j}. \end{eqnarray*}

The species-specific occurrence and detection probabilities are related by a community-level hierarchical component that assumes each probability parameter (e.g., *α*_*i*_*PUBLIC*_*j*_) arises from a common distribution. Model parameters were estimated using a Bayesian analysis of the model with naive prior distributions for coefficients (Normal[*μ* = 0, *σ*^2^ = 1, 000]) and variance of random effects (Gamma[*α* = 0.1, *β* = 0.1]) as there was no previous information on grassland bird occurrence at this spatial scale. We used JAGS (Plummer, 2003) using the R2jags package in R (R Development Core Team, 2013). We ran 4 Markov chain Monte Carlo chains of 20,000 iterations each with the first 1,000 discarded for burn-in. We used the Gelman–Rubin R statistic to assess chain convergence (Gelman & Rubin, 1992).

To assess the fit of data to our model we conducted a posterior predictive check by calculating a Bayesian *p*-value. Specifically, we calculated deviance for each MCMC iteration (*s*) following methodology from Broms, Hooten & Fitzpatrick (2016). However, we calculated deviance for each species using the observed,

\begin{eqnarray*}{D}_{i}^{(s)}=-2\sum _{j=1}^{J}\log \nolimits ({x}_{i,j}{|}{\psi }_{j}^{(s)},{p}_{j}^{(s)}) \end{eqnarray*}

and predicted data (${\tilde {x}}_{i,j,k}\sim Bern[{z}_{i,j}{p}_{i,j,k}];where~{z}_{i,j}\sim Bern[{\psi }_{i,j}]$).

\begin{eqnarray*}{\tilde {D}}_{i}^{(s)}=-2\sum _{j=1}^{J}\log \nolimits ({\tilde {x}}_{i,j}{|}{\psi }_{j}^{(s)},{p}_{j}^{(s)}). \end{eqnarray*}

We calculated a Bayesian *p*-value as the proportion of MCMC samples in which observed deviance was greater than that calculated using predicted data for each species. Generally, a model has poor fit to the data if the *p*-value is >0.95 or <0.05 (Broms, Hooten & Fitzpatrick, 2015).

Lastly, we developed a predictive map of occurrence across the study area by applying species-specific coefficient estimates to spatial covariate layers. Individual species occurrence predictions were summed across cells to generate single a spatial prediction of species richness.

## Results

Within our 413 sampling plots, a total of 36,308 observations of the 20 prairie bird species were recorded over the four years. Species in our grassland guild dominated our observations (89.6%—32,537 of 36,308 observations). Western meadowlark (*Sturnella neglecta*) was the species observed most often, while mountain plover (*Charadrius montanus*) was the least observed. Brewer’s sparrow (*Spizella breweri*) comprised 61.1% (2,303 of 3,771 observations) of the sagebrush guild observations. Grassland comprised 43.3% of the total habitat within the 413 sampling plots, sagebrush made up 26.6% of the habitat, and the remaining 29.8% was classified as “other” habitat (Table 1). Land ownership by public agencies and private landowners was similar: 48.4% and 51.6% respectively (Table 1). The latitudinal gradient based on the centroid of each sample plot ranged from 36.93° to 48.95° (Table 1).

Our measures of environmental associations—land ownership, habitat, and latitude - were important in predicting occupancy for most species (Table 2). Species in the sagebrush guild showed a strong association with the percentage of sagebrush habitat in the plot. Several grassland guild species were positively associated with sagebrush habitat (Table 2). Associations with land ownership were more equivocal than habitat and were mixed among positive and negative for the individual species (Table 2). Land ownership was important in explaining variation in occupancy for 35% of species studies, suggesting that sampling both ownerships is important for understanding patterns of avian occurrence. Most species were positively associated with latitude. Two sagebrush obligates, sage sparrow (*Artemisiospiza nevadensis*) and sage thrasher (*Oreoscoptes montanus*), and two grassland species, Cassin’s sparrow (*Peucaea cassinii*) and mountain plover, were negatively associated with latitude. Grassland or sagebrush landcover were important predictors for detection probability across nearly all species (18/20), though direction and magnitude of coefficients varied by species (Table 3), underscoring the importance of modeling heterogeneity in the observation process.

**Table 3:** Coefficient estimates (and 95% credible intervals) for covariate model on detection probability in multi-species occupancy model. Covariates included the proportion of grassland (Grass) and sagebrush (Sage) landcover and latitude within 1 km^2^ sampling plots. Total number of observation (Obs) for each species is also included as well as Bayesian *p*-values for each species.

Species	Obs	Grass	Sage	Latitude	Intercept	Bayesian *p*-value
Baird’s Sparrrow	277	0.52 (0.33, 0.73)	−0.57 (−0.98, −0.2)	0.14 (−0.16, 0.45)	−1.3 (−1.79, −0.81)	0.44
Bobolink	457	0.43 (0.25, 0.62)	−0.48 (−0.85, −0.15)	−0.01 (−0.37, 0.36)	−0.62 (−1.07, −0.19)	0.59
Cassin’s Sparrow	1,010	−0.11 (−0.22, 0)	−0.01 (−0.74, 0.67)	−0.82 (−1.05, −0.6)	−1.03 (−1.58, −0.51)	0.45
Chestnut-collared Longspur	789	0.84 (0.67, 1.02)	0.08 (−0.13, 0.29)	0.12 (−0.09, 0.33)	−0.76 (−1.08, −0.45)	0.25
Dickcissel	132	−0.48 (−0.9, −0.09)	−0.17 (−0.88, 0.51)	0.48 (−0.15, 1.18)	−0.16 (−0.73, 0.4)	0.48
Grasshopper Sparrow	3,097	0.12 (0.05, 0.2)	−0.44 (−0.54, −0.34)	0.37 (0.3, 0.44)	−0.2 (−0.28, −0.13)	0.37
Horned Lark	6,067	−0.12 (−0.17, −0.06)	−0.31 (−0.36, −0.26)	−0.15 (−0.19, −0.1)	0.53 (0.48, 0.57)	0.01
Lark Bunting	3,972	−0.14 (−0.21, −0.07)	0 (−0.08, 0.08)	−0.14 (−0.2, −0.07)	0.5 (0.44, 0.57)	0.08
Long-billed Curlew	253	−0.18 (−0.4, 0.04)	−0.25 (−0.51, 0)	0.08 (−0.11, 0.28)	−1.78 (−2.08, −1.49)	0.27
Marbled Godwit	204	0.17 (−0.06, 0.39)	0.11 (−0.21, 0.4)	−0.43 (−0.9, 0.01)	−0.81 (−1.5, −0.09)	0.28
McCown’s Longspur	422	0.14 (−0.01, 0.29)	0.23 (0.04, 0.41)	0.31 (0.18, 0.44)	−0.43 (−0.6, −0.26)	0.28
Mountain Plover	52	−0.46 (−1.03, 0.09)	−0.16 (−0.92, 0.56)	0.32 (−0.22, 0.91)	−2.38 (−2.99, −1.84)	0.44
Savannah Sparrow	512	−0.38 (−0.52, −0.25)	−0.33 (−0.51, −0.16)	0.1 (−0.03, 0.23)	−0.87 (−1.06, −0.68)	0.30
Sprague’s Pipit	211	0.13 (−0.08, 0.34)	0.25 (0.01, 0.5)	0.56 (0.09, 1.08)	−1.69 (−2.54, −0.94)	0.50
Upland Sandpiper	953	−0.21 (−0.36, −0.06)	−0.24 (−0.4, −0.09)	−0.64 (−0.86, −0.41)	−0.52 (−0.71, −0.33)	0.20
Vesper Sparrow	3,018	−0.11 (−0.19, −0.04)	0.24 (0.18, 0.31)	0.47 (0.4, 0.54)	−0.62 (−0.7, −0.54)	0.17
Western Meadowlark	11,111	0.36 (0.3, 0.42)	0.11 (0.06, 0.17)	0.28 (0.23, 0.33)	1.58 (1.53, 1.63)	0.44
Brewer’s Sparrow	2,303	−0.2 (−0.46, 0.05)	−0.01 (−0.11, 0.08)	−0.43 (−0.65, −0.23)	−0.64 (−0.9, −0.39)	0.27
Sage Sparrow	712	−2.18 (−3.08, −1.35)	−0.04 (−0.15, 0.07)	−0.39 (−0.85, 0.05)	−2.16 (−3.04, −1.35)	0.36
Sage Thrasher	756	−0.22 (−0.32, −0.12)	0.37 (0.3, 0.44)	−0.01 (−0.09, 0.06)	−0.53 (−0.61, −0.45)	0.11

**Figure 2:**
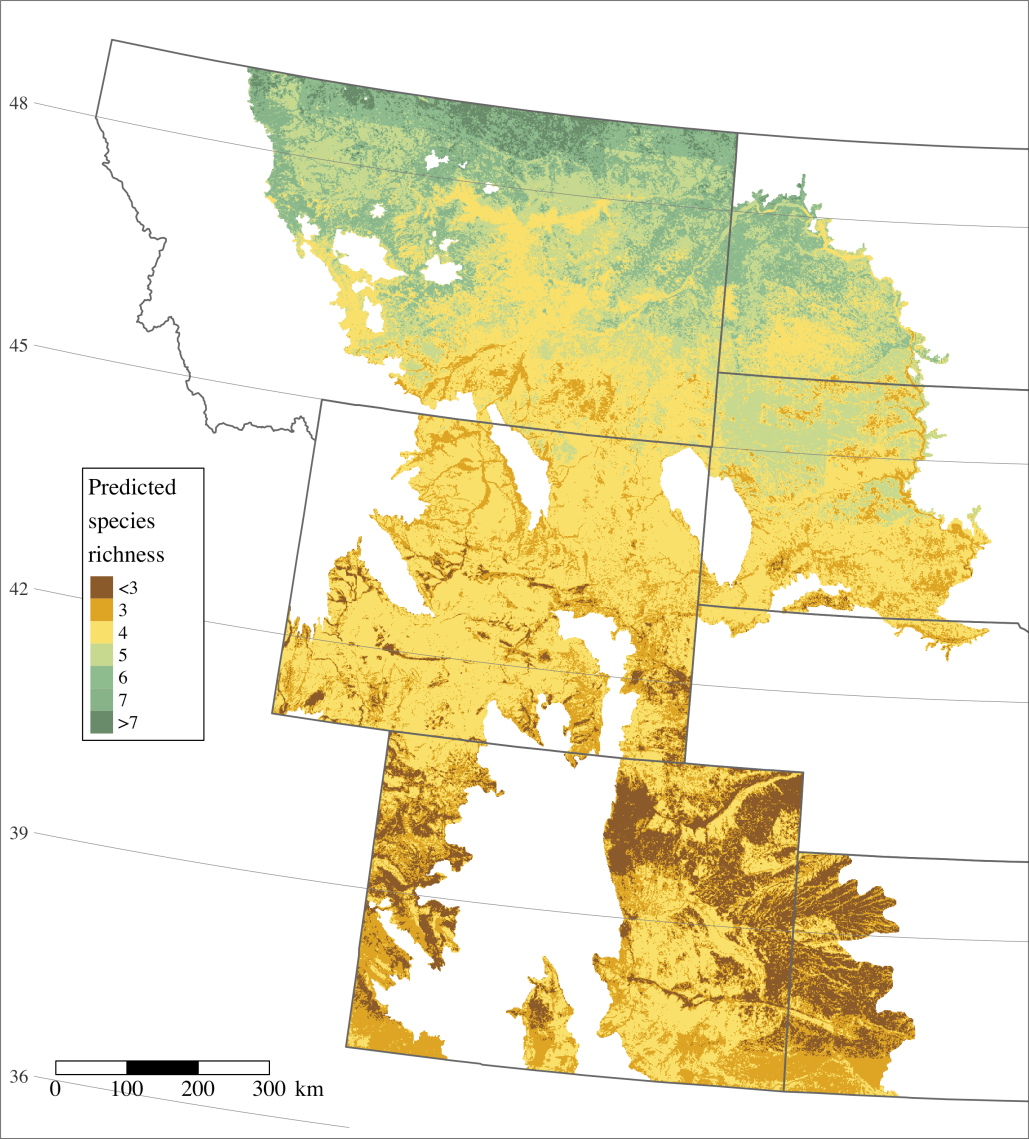
Predicted species richness of 17 grassland and three sagebrush-obligate birds across the study area incorporating six US Bird Conservation Regions and seven states. The spectrum of coloring follows dark green representing higher species richness to dark brown representing lower species richness.

Nineteen of 20 species had a Bayesian *p*-value’s with adequate model fit, while only the horned lark produced a *p*-value suggesting the model did not adequately represent the data (*p* = 0.01; Table 3). Predicted species richness was highest in the northern portion of our study area and declined to the south (Fig. 2), ranging from approximately two to eight species. Twelve of 20 species were significantly and positively associated with latitude while only 4 species demonstrated significant negative associations (Table 2).

## Discussion

Effective conservation planning requires population-level information at the scale of land management that occurs across vast extents (e.g., >10,000 km^2^) and often over long time periods (e.g., multiples years via BLM landscape approach, USFS land and resource management plans, State Wildlife Action Plans). Yet often lacking are large-scale monitoring programs that employ a consistent methodology and a probabilistic-sampling design necessary for robust inference at regional scales. The IMBCR program provides a large-scale dataset that adheres to design and survey principles allowing for unbiased estimation of critical state variables (e.g., occupancy) that transcends ecotype and political boundaries. In particular, multiple surveys within randomly-sampled grids allows for an explicit model-based treatment of the observation process which often confounds ecological data. This study exemplifies how these data can provide community-level inference across broad scales for use in the ecology and management of prairie birds.

We chose a guild approach here to broaden our inference across species as well as space. The guild approach complements single-species approaches of analyzing data from the IMBCR program (Pavlacky et al., 2012). Analyses of guilds provide information on factors influencing species richness while single-species analyses can look more deeply at habitat relationships for target species. Guilds also have the potential to better reflect the level to which ecosystem services remain intact. At the species level, habitat associations influencing the probability of occupancy varied, though a few general patterns emerged. First, sagebrush guild birds are more directly associated with sagebrush habitats. The absence of sagebrush habitat led to low probabilities of occupancy for those species, similar to others’ findings (Knick et al., 2003; Donnelly et al., 2016). On the other hand, grassland obligates showed broader vegetative associations, evidenced by a negative association of grasslands for one species (dickcissel) and equivocal relationships for five species (Table 2). This finding may reflect a broader range of habitats, including our definition of grassland and sagebrush habitat, that can provide the ecological resources required by grassland obligates. Whereas sagebrush guild species may exclusively require sagebrush habitat.

The variation in the strength of habitat associations between avian guilds may have important management implications. For example, an association with sagebrush habitat was significantly positive for vesper sparrow (*Pooecetes gramineus*), a species considered to be a grassland obligate (Vickery et al., 1999) that had no significant association with grassland in the study. Thus, conservation efforts targeting sagebrush habitat may not only have positive effects for sagebrush obligates, but also for vesper sparrows and, to a lesser extent, horned larks (*Eremophila alpestris*) and lark buntings (*Calamospiza melanocorys*). Second, more species in the grassland guild tended to be associated with more northern latitudes. Only two grassland species, the Cassin’s sparrow (*Peucaea cassinii*) and mountain plover, and two sagebrush were strongly associated with more southern latitudes. Patterns in species occupancy as a function of land ownership were highly variable, as evidenced by varying coefficient estimates. This variability is likely driven by a number of factors. For instance, on public lands, land uses vary with different administrative mandates (e.g., federal agency multiple use mandates) or land designations (e.g., wilderness). On private lands, there is greater diversity in the types of land use (e.g., including multiple types of agricultural production) than on public lands. Thus, effective conservation strategies must transcend land tenure to confer community-level benefits. Future analyses of IMBCR data, or similarly robust sampling efforts, could evaluate associations between bird occupancy and ownership in finer categories, such as specific agency or tribal jurisdiction, or by land designation.

The area of highest richness in grassland and sagebrush obligate birds considered in this study was the most poleward region, northern Montana and western North Dakota, a landscape which is seeing continued large-scale land conversion for oil and gas development and agricultural tillage. Map-based predictions of species richness offer a spatially-explicit targeting tool for conservation in light of expanding land conversion. In particular, maps used in concert with science-based products for the anticipated growth of agriculture and energy development (Copeland et al., 2009) offer a template for conservation planning to avoid and minimize major known stressors to prairie birds. Whereas biological planning often relies on small-scale studies of focal species, science from the IMBCR offers a community-level product at the scale of management plans.

Our finding of increasing species richness with latitude provide an interesting contrast to results from most studies on latitudinal-diversity gradients. Only a few other studies have found a similar reverse relationship between avian species richness and latitude (Rabenold, 1979; Karanth et al., 2014). One explanation for this pattern is that we explicitly examined grassland- and sagebrush-obligate bird species during the breeding season. These species are inextricably linked to the existence of relatively predictable pulses of primary production and arthropod biomass in a relatively short growing season in more northern regions (Rabenold, 1979). Such predictable, superabundant food could permit the coexistence of similar species (Wiens, 1974). Additional mechanisms leading to our study’s disparity with the latitudinal biodiversity gradient theory are likely abiotic factors, such as soil moisture (Hillebrand, 2004; Mittelbach et al., 2007), and warrant further consideration.

Examining biological diversity at the landscape-scale provides insights not available from focal-scale studies. The larger spatial extent of these studies demonstrates the importance of habitat diversity in maintaining complete guilds of species, while delineating areas of high co-occurrence of prairie birds. Focal-scale analyses can document what is present in a study area, but landscape-scale studies place local biodiversity in the context of an entire region, or perhaps breeding range, while simultaneously highlighting the value of local areas.

## Additional Information and Declarations

### Data Availability

The following information was supplied regarding data availability: The research in this article did not generate any raw data. Data was used from Integrated Monitoring in Bird Conservation Regions (IMBCR) and we do not own this data. This is referenced in the methods, and the interested reader can quickly find reference to this program online (http://www.birdconservancy.org/what-we-do/science/monitoring/imbcr-program/).
